# The Water-Soluble [60]Fullerene Derivatives as Binders of SARS-CoV‑2 Proteins

**DOI:** 10.1021/acsmedchemlett.6c00282

**Published:** 2026-08-03

**Authors:** Katarzyna Malarz, Tainah Dorina Marforio, Robert Gawecki, Dominik Dreszer, Matteo Calvaresi, Maciej Serda

**Affiliations:** † Institute of Physics, 431559 University of Silesia in Katowice, Katowice 40-007, Poland; ‡ Department of Chemistry “Giacomo Ciamician”, University of Bologna, 40126 Bologna, Italy; § Institute of Chemistry, University of Silesia in Katowice, Katowice 40-007, Poland

**Keywords:** [60]fullerenes, SARS-CoV-2, nanomaterial inhibitors, 2019-nCoV spike, PLPro, 3CL-Mpro, RNA-dependent RNA polymerase

## Abstract

Water-soluble fullerene derivatives represent emerging molecular nanomaterials for antiviral drug discovery, yet their target selectivity against SARS-CoV-2 proteins remains insufficiently defined. Here, four functionalized [60]­fullerenes, C_60_(OH)_24_, C_60_ser, C_60_ALA, and C_60_Trp, were evaluated as binders of key SARS-CoV-2 targets involved in viral entry and replication: spike glycoprotein, papain-like protease, 3CL main protease, and RNA-dependent RNA polymerase. Microscale thermophoresis revealed distinct target-dependent binding profiles governed by fullerene functionalization. The C_60_Trp fullerene was the most active derivative, binding Spike with nanomolar affinity (K_d_ = 94.8 nM) and showing micromolar affinity toward papain-like protease and 3CL main protease. Docking and molecular dynamics simulations supported the preferential interaction of C_60_Trp with the spike protein cavity through combined fullerene cage complementarity and polar interactions with lysine and arginine residues. The tested derivatives showed low cytotoxicity in A549 cells, and C_60_Trp was efficiently internalized. These findings identify tryptophan-functionalized [60]­fullerene as a promising multivalent scaffold for SARS-CoV-2 protein targeting.

## Graphical abstract

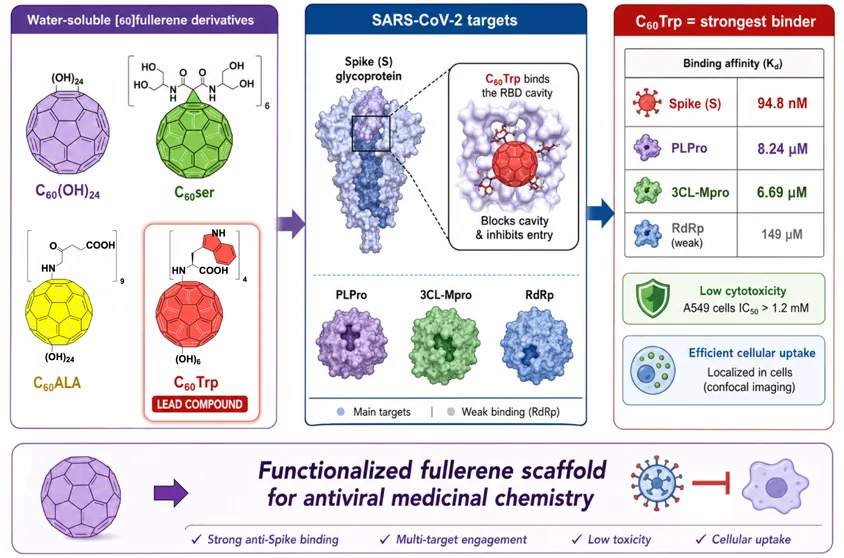

Viruses remain a persistent global health challenge, prompting the search for innovative antiviral strategies, often based on novel types of scaffolds, even macrocyclic or at the nanometric level.^1^ One emerging approach involves the creation of functionalized fullerene derivatives, which have demonstrated broad-spectrum antiviral activity against diverse viruses, including human immunodeficiency virus (HIV), herpes simplex virus, influenza, Ebola, cytomegalovirus, and even coronaviruses.^2–4^ These findings position *antiviral buckyballs* as promising pharmacophores in medicinal chemistry, as fullerenes are convenient substrates for chemical functionalization, like Bingel-Hirsch cyclopropanations and azide–alkyne click chemistry,^5–7^ working as the bridge between nanomaterials and small molecules. Mechanistically, fullerenes exert antiviral effects through several molecular pathways.^8^ Owing to their hydrophobic, π-conjugated surfaces, fullerenes can bind strongly into the hydrophobic pockets of viral proteins, such as the main protease of the SARS-CoV-2 virus Mpro, thereby inhibiting their function.^9^ Fullerene antivirals may also modulate host factors; for instance, a polyhydroxylated [60]­fullerene showed pronounced antioxidant activity in Zika-infected cells and reduced Zika virus production by ∼90%, indicating an indirect mechanism via mitigation of virus-induced oxidative stress.^10^ These examples underscore the versatility of fullerene-based antivirals, which can be tailored to engage viral targets for SARS-CoV-2 infection. Recently, Katagishi and co-workers investigated the inhibitory potential of several fullerene derivatives against the SARS-CoV-2 main protease. The lead candidate was a polycarboxylic, malonic acid–type fullerene derivative that reduced protease activity by 50% at a concentration of 0.20 μM.^11^ In 2023, Page et al. assessed the *in vitro* anti–SARS-CoV-2 activity of fullerene-based materials functionalized with linear polyglycerol amine sulfate or dendritic polyglycerol sulfate.^12^ Interestingly, recent studies revealed that novel carboxylated [60]­fullerene nanomaterials (derived from the chlorofullerene C_60_Cl_6_) were shown to inhibit SARS-CoV-2 infection *in vitro*, with half-maximal inhibitory concentrations (IC_50_) in the submicromolar range (∼0.39 μM) and high selectivity indices.^13^

The recently published computational studies consistently show that pristine fullerenes can bind tightly in the SARS-CoV-2 main protease (Mpro) active site.^9^ Docking and molecular dynamics (MD) simulations reveal that a C_60_ fullerene snugly occupies the substrate-binding pocket, overlapping the position of known Mpro inhibitors. The spherical buckyball cage displays strong shape complementarity and forms multiple interactions with the pocket: it stacks against the catalytic His41 residue (π–π sandwich-like contact) and makes hydrophobic contacts with surrounding residues. In contrast, while the fullerene can fit into the papain-like protease (PLPro) binding cleft, the pocket may accommodate the rigid C_60_ a bit less snugly than Mpro does.^14^ Beyond direct blocking, functionalized fullerenes can inactivate spike by allosterically “locking” its conformation. Interestingly, more recent publications have shown that preincubation of human nasal epithelial cells (HNEpCs) with the fullerenol-ectoine composite for 1 h protected them against the Spike protein—probably by preventing the Spike protein–host cell receptor angiotensin-converting enzyme 2 (ACE2) interactions.^15^

The therapeutic versatility of engineered water-soluble fullerene derivatives has been previously reported by our group, particularly regarding their roles in enzyme inhibition and photodynamic applications.^16,17^ Data suggest that appropriate functionalization is a critical determinant for cellular uptake and organelle-specific targeting, allowing for the precise modulation of molecular pathways.^18,19^ Hence, to verify the usefulness of water-soluble derivatives of [60]­fullerene as inhibitors of SARS-CoV-2 proteins, we synthesized four different types of buckyballs, based on different synthetic approaches and functionalization techniques. Among water-soluble fullerene derivatives, C_60_(OH)_24_ remains the most extensively characterized example. Synthesized from the brominated intermediate, its structural profile allows for significant noncovalent interactions with proteins, specifically through the synergistic effects of electrostatic attraction and hydrogen bond formation.^20^ The investigation also included the T_*h*_ symmetrical hexakisadduct C_60_ser to represent the broader family of neutral malonic acid derivatives with documented antiviral activity.^4^ Additionally, two bifunctional amino acidic derivatives of [60]­fullerene (C_60_ALA and C_60_Trp) were selected to explore the synergy/antagonism between carboxyl and hydroxyl groups on the fullerene cage and viral protein inhibition. Our previous reports identify C_60_ALA as a nontoxic prodrug of protoporphyrin IX, while C_60_Trp has been used specifically as a metal-chelating agent.^21,22^ The chemical structures of the test compounds used in the current study are shown in Figure 1. The synthesis of fullerene derivatives was previously described in our reports, and we used the same batches of nanomaterials as before.^21–23^ The C_60_ser fullerene is a structurally discrete T_*h*_-symmetric hexakisadduct, and the hydroxyfullerene C_60_(OH)_24_ was synthesized in a two-step procedure from C_60_Br_24_ to avoid the formation of complex sodium adducts and the need for a phase-transfer catalyst. In contrast, C_60_ALA and C_60_Trp are materials with distributions in the number and/or position of surface addends. Their formulas are therefore used only to denote nominal average compositions confirmed by elemental analysis and mass spectrometry.

**Figure 1:**
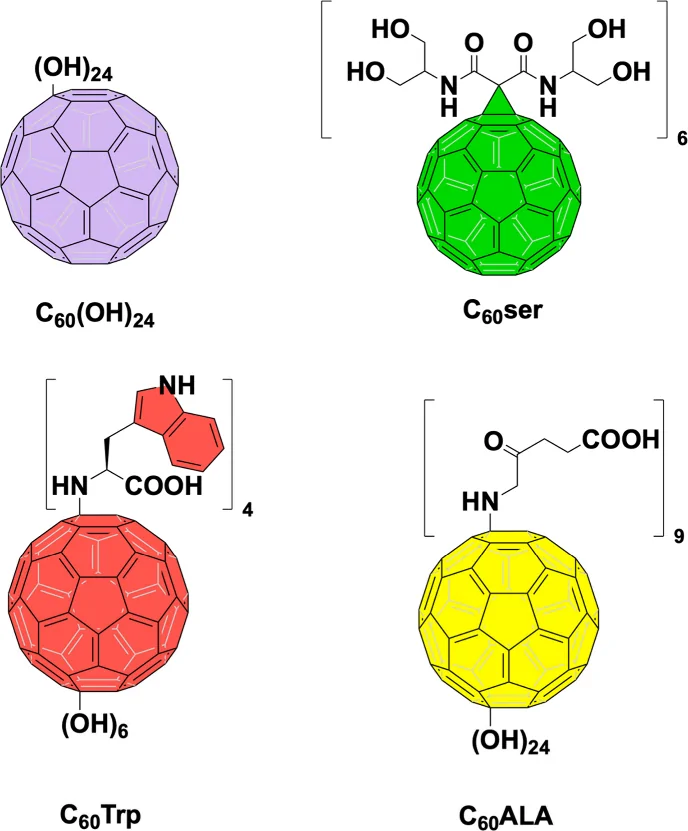
Chemical structures and synthetic pathways for the creation of the four studied water-soluble fullerene derivatives C_60_(OH)_24_, C_60_ser, C_60_Trp, and C_60_ALA.

Rather than representing an exhaustive SAR series, the four water-soluble fullerene materials were selected as a deliberately compact panel of chemical diversity. Hydroxyfullerene was included as a benchmark with a densely hydroxylated, highly hydrated surface; the T_*h*_-symmetric C_60_ser hexakisadduct represented a discrete and sterically defined neutral multivalent architecture; and the C_60_ALA/C_60_Trp nanomaterials compared two amino-acid-derived, carboxylate-containing fullerene surfaces that differ principally in flexibility and aromaticity. C_60_ALA presents flexible aliphatic groups, whereas C_60_Trp introduces indole rings capable of additional hydrophobic, aromatic, and π – π interactions. The panel was therefore designed to test how hydroxyl density, addend symmetry, charge/polarity, flexibility, and aromaticity influence binding to viral proteins involved in entry, proteolysis, and genome replication.

Water-soluble [60]­fullerene derivatives possess an outer-hydrophilic/inner-hydrophobic architecture and therefore exhibit physicochemical properties distinct from those of amphiphilic molecules with other molecular geometries. Their tendency to form relatively large aggregates is generally attributed to the highly hydrophobic fullerene core.^24^ Previous studies have reported average hydrodynamic diameters of fullerene aggregates ranging from 100 to 250 nm, which is consistent with the DLS results obtained in the present study (135 nm and zeta potential −45 mV, see Supporting Information, Figures S1 and S2).^17,25^ However, the dynamic character of the aggregation process has received comparatively little attention. In addition to being concentration-dependent, the aggregation of water-soluble fullerenes is influenced by pH, salt concentration, and the presence of serum proteins.

The next step in our studies was to study the interactions of the tested compounds with SARS-CoV-2 proteins: PLPro and Mpro proteases, spike protein, and RNA-dependent RNA polymerase (RdRp). The selected protein targets cover the entire life cycle of the virus, from its initial entry to intracellular replication.^26^ The first critical component is the Spike protein, which binds to human ACE2 and other coreceptors, facilitating viral entry via host cell membrane fusion. The next two targets, the PLPro and 3CL-Mpro viral proteases, are responsible for the proteolytic cleavage of two large polyproteins (pp1a and pp1ab) into functional nonstructural proteins essential for viral replication within the host cell.^27^ Finally, the RdRp, acting as the core of the replication-transcription complex (RTC), coordinates the synthesis of new viral RNA genomes capable of infecting subsequent cells.^28^ The binding affinity profiles and dissociation constants (K_d_) of the fullerenes toward the tested SARS-CoV-2 proteins were determined using microscale thermophoresis (MST). After fluorescent labeling of the target proteins with His-tag or lysine-specific dyes, they were incubated with a series of dilutions of fullerenes and corresponding inhibitors to determine the bound and unbound fractions quantitatively. The K_d_ values are presented in Table 1, and the binding curves for the most potent derivatives against PLPro, RdRp, spike protein, and 3CL-Mpro protease are shown in Figure 2. The rest of the binding profiles are given in Figures S1–S4.

**Table 1:** Binding Constants (K_d_ Values) Measured for Fullerenes and Reference Compounds against Tested Covid-Proteins

	Binding affinity K_d_ (μM)			
Compounds	PLPro	RNA-dependent RNA polymerase (RdRp)	2019-nCoV spike	3CL-Mpro
C_60_(OH)_24_	36.97 ± 3.08	183.4 ± 4.0	NB^a^	10.71 ± 0.30
C_60_ser	37.92 ± 3.35	15.23 ± 5.34	10.38 ± 1.84	NB
C_60_ALA	19.83 ± 3.49	24.85 ± 2.60	18.85 ± 0.07	NB
C_60_Trp	8.24 ± 0.95	149.3 ± 6.4	0.0948 ± 0.0155	6.69 ± 0.32
dasatinib	3.97 ± 0.80			
H69C2			0.0947^b^	
EIDD-1931		1.16 ± 0.20		
nirmatrelvir				0.006 ± 0.001

a NB: nonbinding.

b Data from ref 29.

**Figure 2:**
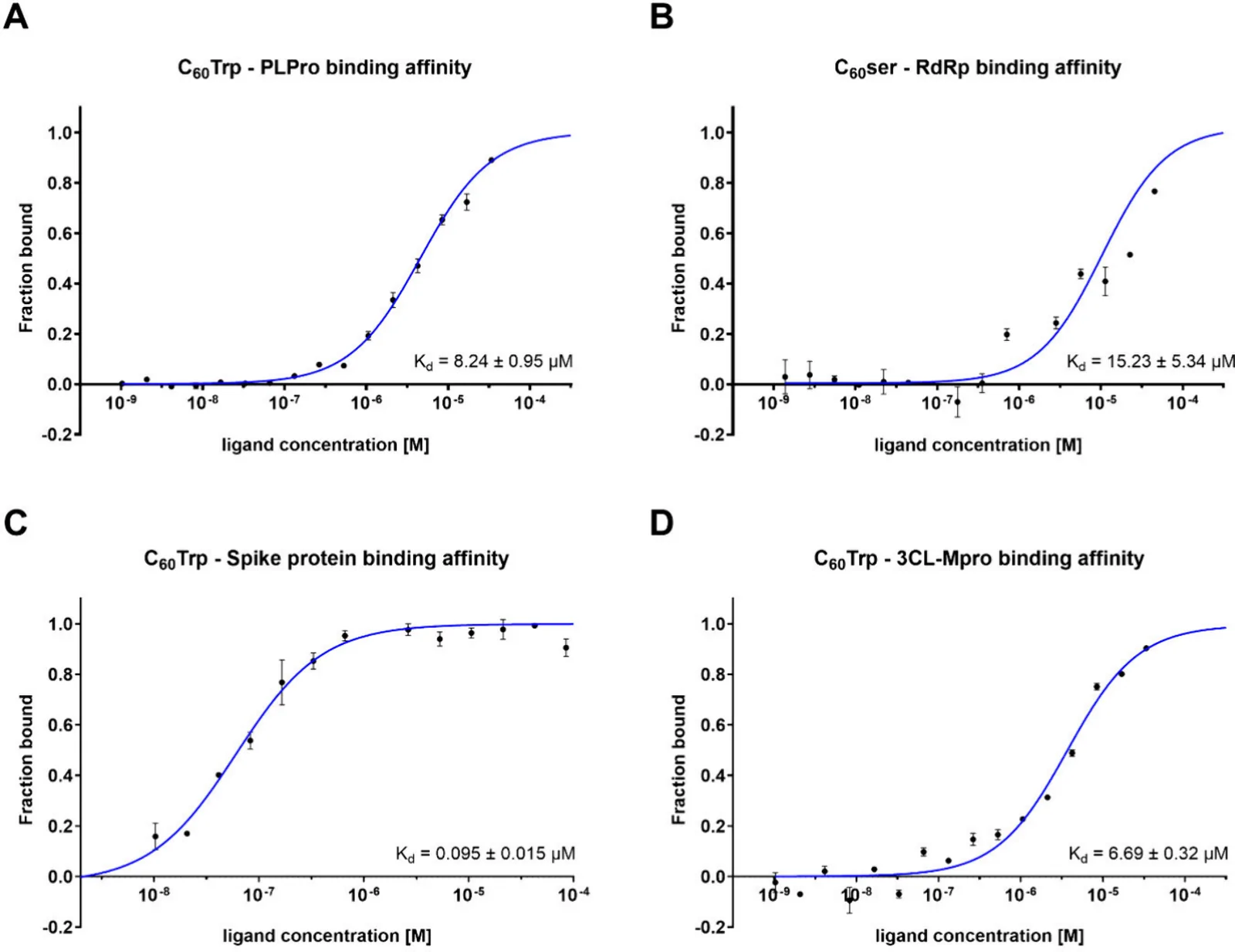
Binding affinity profiles of the most potent fullerenes toward SARS-CoV-2 target proteins. Representative microscale thermophoresis (MST) titration curves showing the interaction of selected functionalized fullerenes with (A) PLPro, (B) RdRp, (C) Spike protein, and (D) 3CL-Mpro. The fraction bound is plotted against the ligand concentration on a logarithmic scale. Each data point represents the mean ± SD of three independent measurements (*n* = 3).

Among the evaluated targets, the Spike glycoprotein emerged as the most susceptible to the tested fullerenes. Notably, C_60_Trp demonstrated a remarkably high binding affinity to the protein with a calculated K_d_ of 94.8 nM. Interestingly, a comparable interaction strength (K_d_ = 94.7 nM, determined via surface plasmon resonance) was previously reported for the characterized flavone derivative H69C2 toward the receptor-binding domain (RBD) of the Spike protein.^29^ Furthermore, this tryptophan-functionalized derivative displayed the strongest interactions with the two tested viral proteases, PLPro and 3CL-Mpro, revealing calculated K_d_ values of 8.24 and 6.69 μM, respectively. However, it showed only a very weak affinity toward the RNA-dependent RNA polymerase (RdRp).

The other functionalized fullerenes exhibited more varied binding profiles. Both C_60_ser and C_60_ALA exhibited favorable binding profiles for the Spike protein, with dissociation constants ranging from 10 to 19 μM. Regarding other COVID-19 targets, C_60_ser showed a good affinity toward RdRp, although it was significantly weaker than that of the well-known reference inhibitor, EIDD-1931. In turn, fullerene, the 5-aminolevulinic acid functionalized, maintained consistent binding behavior across the Spike protein, PLPro, and RdRp. Importantly, neither C_60_ser nor C_60_ALA exhibited measurable binding to the Mpro protease. Conversely, C_60_(OH)_24_ exhibited a distinct profile relative to the tested proteins. It showed moderate to good capacity to interact with the 3CL-Mpro and PLPro proteases (K_d_ of 10.7 and 37 μM, respectively), without showing significant affinity for the Spike protein or RdRp.

In an attempt to establish a preliminary structure–property relationship between the different fullerene derivatives and their diverse biological activity, we considered how their structural features may influence protein recognition and binding affinity.^30,31^ Fullerenol possesses a densely hydroxylated and strongly hydrated shell that can support hydrogen bonding but also masks much of the cage’s hydrophobic π-surface; this may account for its lack of measurable Spike binding and its more modest protease affinity. The discrete Th-symmetric C_60_ser hexakisadduct presents six spatially defined, neutral, diserinol malonate arms. This architecture may be better accommodated by broad, solvent-exposed protein surfaces, while its steric coverage may disfavor entry into the narrower 3CL-Mpro pocket. C_60_ALA fullerene contains flexible carboxylate-bearing aliphatic groups and displays moderate binding to several targets, consistent with electrostatic and hydrogen-bonding contributions but without an additional aromatic recognition element. The C_60_Trp combines a relatively exposed fullerene scaffold with carboxylate/hydroxyl groups and indole rings. This arrangement can simultaneously support cage-driven hydrophobic complementarity, electrostatic contacts with basic residues, hydrogen bonding, and aromatic or cation−π interactions, providing a plausible rationale for its stronger binding to Spike, PLPro, and 3CL-Mpro.

Docking studies depicted in Figure 3 have shown that fullerene derivatives tend to preferentially bind to the catalytic sites/substrate-binding pockets of the enzymes PLPro, RdRp, and 3CL-Mpro, as well as to the receptor-binding domain (RBD) of the Spike protein. Concave–convex complementarity plays a key role in the binding of fullerenes and their derivatives to proteins, explaining their general behavior as inhibitors. A focus was placed on the interaction between C_60_Trp and the Spike glycoprotein, as this case experimentally shows the highest binding affinity among those studied. Starting from the best docking pose, molecular dynamics simulations were performed, followed by MM-GBSA binding energy analysis (Figure 4A-C). Whereas for pristine fullerenes the binding is mainly driven by π–π and hydrophobic interactions with nonpolar residues, in fullerene derivatives additional interactions become crucial, such as hydrogen bonds and salt bridges when net charges are present. Indeed, the residues showing the strongest interactions with C_60_Trp include Lys964.C and Lys304.C, as well as Arg765.A, which primarily interact with the carboxylate groups and hydroxyl functionalities of C_60_Trp rather than with the carbon cage itself. Nevertheless, the fullerene cage plays a primary role in conferring rigidity and a well-defined geometry to these chemical moieties.

**Figure 3:**
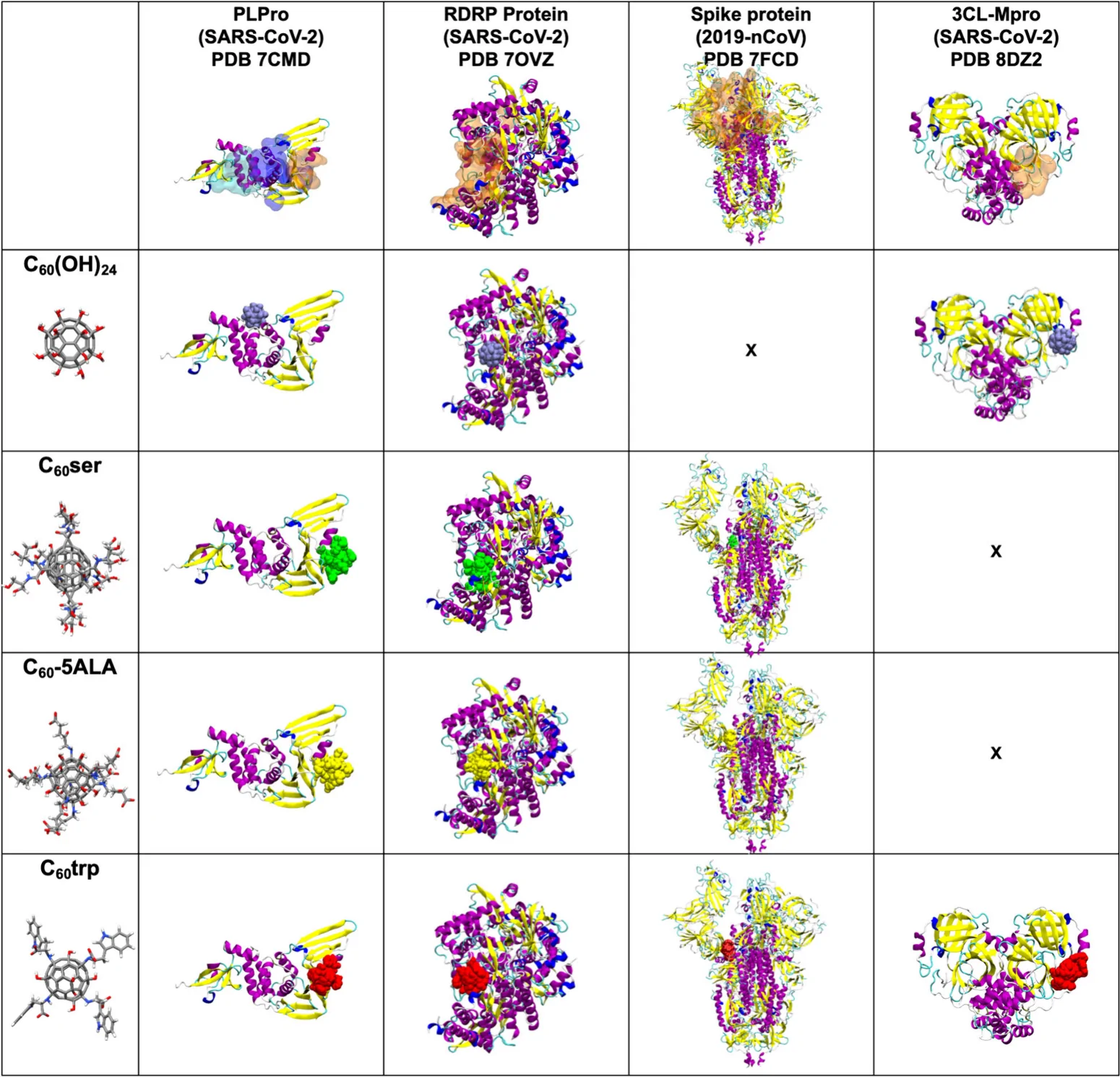
Most interacting binding poses of different fullerene derivatives (C_60_(OH)_24_, C_60_ser, C_60_-ALA, and C_60_Trp) in complex with selected SARS-CoV-2 proteins: PLPro, RNA-dependent RNA polymerase (RdRp), Spike protein, and 3CL-Mpro. PLpro (SARS-CoV-2) (PDB= 7CMD) active site binding pocket (light blue), Sub1 binding pocket (blue) and S3/S4 binding pocket (orange). RDRP protein (SARS-CoV-2) (PDB = 7OVZ), RNA binding pocket (orange). Spike protein (2019-nCoV) (PDB = 7FCD) RBD binding pocket (orange). 3CL-Mpro (SARS-CoV-2) (PDB = 8DZ2) substrate binding pocket (orange).

**Figure 4:**
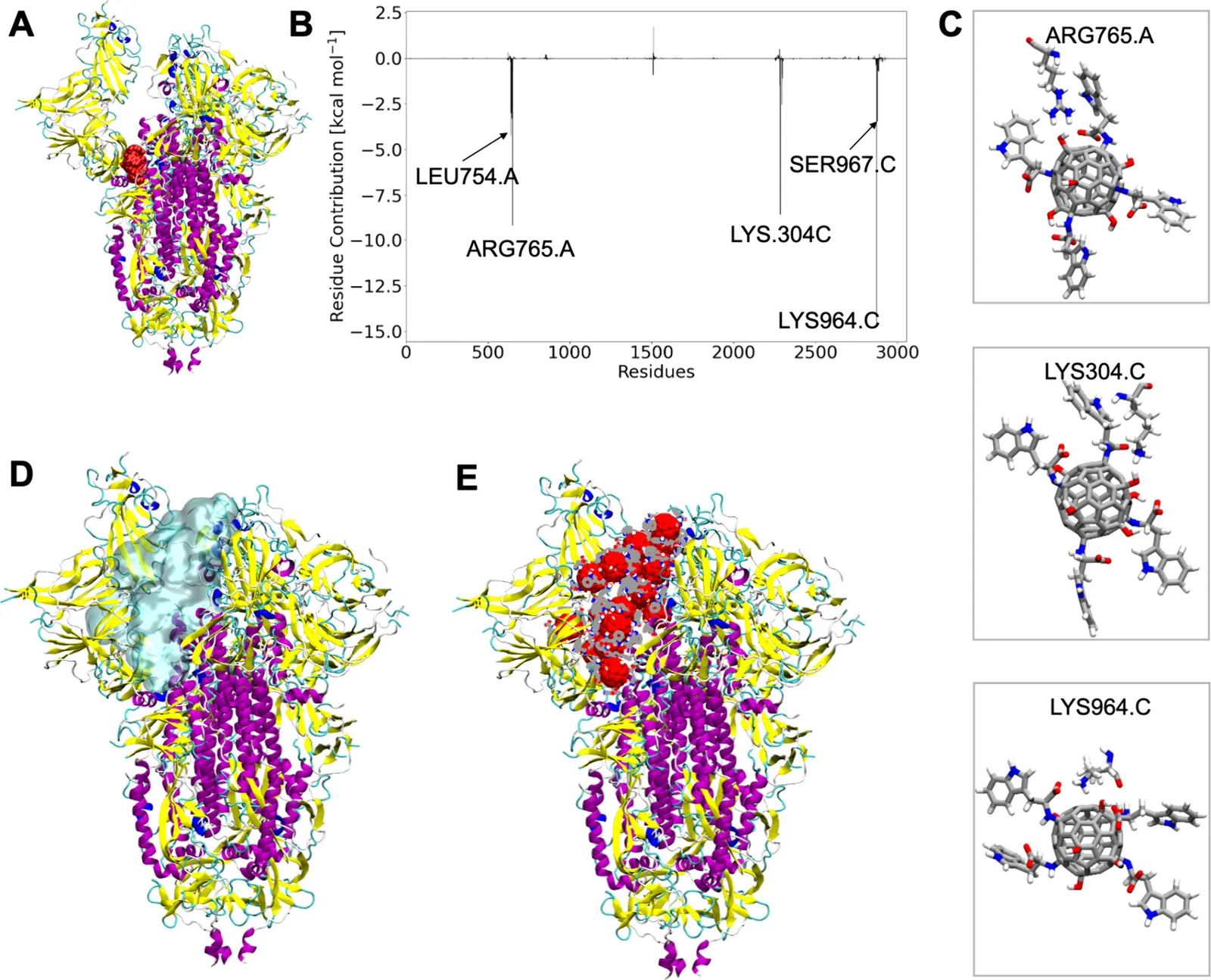
(A) Interaction between C_60_trp and the Spike protein. (B) Per-residue decomposition of the binding free energy (ΔG_binding_). (C) Representative interactions between C_60_Trp and key residues: Arg765.A (top), Lys304.C (middle), and Lys964.C (bottom). (D) Binding cavity of the Spike protein. (E) Multiple occupancy of C_60_trp molecules within the Spike protein binding cavity.

The trimeric Spike protein mediates viral entry through its S1 subunit, which recognizes the ACE2 receptor *via* a conformationally dynamic receptor-binding domain (RBD). The RBD alternates between closed and open states, the latter exposing a binding cavity (∼50 Å in width and ∼65 Å in depth) that is essential for ACE2 interaction.

Small-sized nanoparticles can effectively inhibit viral infection by occupying this cavity within the Spike trimer and interacting with the RBD.^32,33^ Considering the hybrid nature of fullerene derivatives, which lie at the interface between molecular and nanoparticulate systems (i.e., “molecular nanoparticles”), multiple C_60_Trp units may sequentially occupy the cavity, thereby inducing conformational inhibition of the Spike protein (Figure 4D-E). However, although the vast dimensions of the Spike protein’s binding cavity could theoretically accommodate multiple ligands, the monovalent dose–response observed in the MST assay indicates a single, primary high-affinity binding event for C_60_Trp, as identified by the MD study.

Recent literature indicates that functionalized fullerenes can maintain or even demonstrate superior inhibitory effects against recent variants (e.g., XBB) compared to the wild-type, likely because fullerene binding relies heavily on the broad structural occlusion of the binding cavity rather than highly specific, easily mutated point contacts.^34^

Further studies focused on assessing the cytotoxicity of the tested fullerene nanomaterials against A549 nonsmall cell lung cancer cells. It is worth noting that A549 cells are considered one of the standard models for screening anti-SARS-CoV-2 drugs due to their high expression of human ACE2 receptors,^35,36^ which mediate SARS-CoV-2 entry and host cell infection. This viral entry is driven by the S1 subunit of the Spike protein, which contains a RBD that directly binds to the extracellular peptidase domain of hACE2 (hACE2-PD) with high affinity.^37^ As shown in Figure 5C, none of the evaluated fullerenes exhibited cytotoxicity against the human host cells, even at high concentrations exceeding 900 μM. In addition, we examined the intracellular accumulation of C_60_Trp, identified as the most active fullerene against SARS-CoV-2 proteins among the compounds tested. Confocal microscopy revealed that C_60_Trp was efficiently internalized and exhibited no apparent organelle-specific localization. Instead, the compound was distributed diffusely throughout the cytoplasm and was also detected in the cell nucleus (Figure 5A, B). Such a widespread intracellular distribution suggests that C_60_Trp can access multiple cellular compartments and may therefore interact with a broad range of intracellular targets. This feature may be particularly relevant for antiviral activity, as SARS-CoV-2 replication and host–virus interactions involve multiple intracellular compartments.

**Figure 5:**
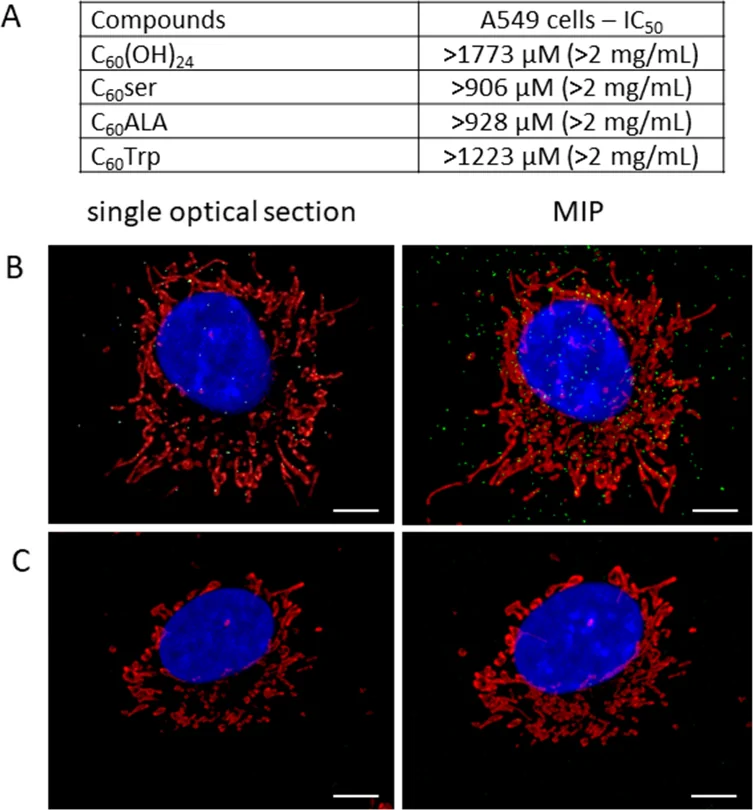
Cytotoxicity profile of analyzed fullerene derivatives and intracellular localization of C_60_Trp. (A) Tabulated summary of cytotoxicity data for the analyzed fullerene derivatives in A549 cells. (B) Representative confocal micrographs of cells treated with C_60_Trp, showing the central optical section and the corresponding maximum intensity projection (MIP) of the acquired z-stack, and (C) untreated control cells. Nuclei are visualized in blue, mitochondria in red, and C_60_Trp in green. Scale bar 10 μm.

## Supplementary Material



## Data Availability

Data will be made available upon request.

## Notes

Materials and methods, size and zeta potential of fullerene nanomaterial C_60_Trp, and binding affinity profiles of fullerenes toward SARS-CoV-2 target proteins (PDF)

## Abbreviations

3CL-Mpro: 3C-like main protease
ACE2: angiotensin-converting enzyme 2
C_60_ALA: 5-aminolevulinic acid-functionalized [60]­fullerene
C_60_(OH)_24_: polyhydroxylated [60]­fullerene (fullerenol)
C_60_ser: T_h_-symmetric diserinol malonate [60]­fullerene hexakisadduct
C_60_Trp: tryptophan-functionalized [60]­fullerene
COVID-19: coronavirus disease 2019
DLS: dynamic light scattering
hACE2: human angiotensin-converting enzyme 2
hACE2-PD: human ACE2 peptidase domain
HIV: human immunodeficiency virus
HNEpCs: human nasal epithelial cells
IC_50_: half-maximal inhibitory concentration
MD: molecular dynamics
MIP: maximum intensity projection
MM-GBSA: molecular mechanics/generalized Born surface area
Mpro: main protease
MST: microscale thermophoresis
NB: no measurable binding
PDB: Protein Data Bank
PLPro: papain-like protease
RBD: receptor-binding domain
RdRp: RNA-dependent RNA polymerase
RTC: replication-transcription complex
SAR: structure–activity relationship
SARS-CoV-2: severe acute respiratory syndrome coronavirus 2
SD: standard deviation
