# Supplementary material for: The Water-Soluble [60]Fullerene Derivatives as Binders of SARS-CoV‑2 Proteins
Source: ACS Med Chem Lett. 2026 Aug 3;17(10):2153–9. doi: 10.1021/acsmedchemlett.6c00282 (PMC13656267; doi:10.1021/acsmedchemlett.6c00282)
Supplement: Supplementary file 1 [file ml6c00282_si_001.pdf]

SUPPORTING INFORMATION

The water-soluble [60]fullerene derivatives as binders of SARS-COV2 proteins

Katarzyna Malarz[1], Tainah Dorina Marforio[2], Robert Gawecki[1], Dominik Dreszer[3], Matteo Calvaresi[2], Maciej Serda[3*]

[1]*Institute of Physics, University of Silesia in Katowice, Poland*

[2] *Department of Chemistry "Giacomo Ciamician", University of Bologna, Italy*

[3]*Institute of Chemistry, University of Silesia in Katowice, Poland*

Email: maciej.serda@us.edu.pl

Materials and methods

EIDD-1931 and nirmatrelvir were purchased from Prospecta. Dasatinib was obtained from Cayman Chemical.

PLPro binding affinity assays

The binding affinities of fullerenes and dasatinib toward SARS-CoV-2 PLPro were determined by microscale thermophoresis (MST) using the Monolith NT.115 Blue/Red device (NanoTemper Technologies, Germany). Recombinant human PLPro protein (His-tagged) (Biomol) was fluorescently labeled using the Protein Labeling Kit RED-NHS 2nd Generation (NanoTemper Technologies). The final labeled protein concentration was 50 nM. Experiments were performed in a buffer containing 50 mM Tris-HCl (pH 7.8), 300 mM NaCl, 10 mM $MgCl_2$, 0.1% Pluronic F-127, 0.1% BSA, and 1 mM EDTA. Stock solutions of fullerenes were prepared in deionized water at a concentration of 2 mg/mL ($C_{60}(OH)_{24}$ = 1773 µM; C_{60}ser = 906 µM; C_{60}ALA = 928 µM; C_{60}Trp = 1223 µM), while dasatinib was dissolved in DMSO to a stock concentration of 8.35 mM. For the MST measurements, the highest concentration in the serial dilution series for each fullerene was prepared by a 20-fold dilution of the respective stock in assay buffer (e.g., 88.7 µM for $C_{60}(OH)_{24}$), followed by 2-fold serial dilutions. Dasatinib was similarly prepared using a starting concentration of 50 µM, followed by 2-fold serial dilutions in the assay buffer to ensure a final DMSO concentration below 1% (v/v) to

maintain protein stability. For Kd determination, labeled proteins were incubated with serial dilutions of tested compounds for 15 minutes at room temperature. Samples were then centrifuged (12,000 rpm for 10 minutes), placed in standard capillaries (NanoTemper Technologies) and measured at 25°C using 80% LED power and 40% MST power. Experiments were repeated at least three times. Data analysis was performed using MO.Affinity Analysis 2.3 software (NanoTemper Technologies). All final graphs were prepared using GraphPad Prism 10.

RNA-dependent RNA polymerase binding affinity assays

The binding affinities of fullerenes and EIDD-1931 toward RNA-dependent RNA polymerase/ RdRp protein (SinoBiological) were determined using MST as described for PLPro, with the following modifications. RdRp was fluorescently labeled using the Monolith His-Tag RED-tris-NTA 2nd Generation labeling kit (NanoTemper Technologies). The assay was conducted in an assay buffer, containing 50 mM HEPES (pH 7.5), 300 mM NaCl, 10 mM $MgCl_2$, and 0.05% Tween 20. EIDD-1931 was prepared from a 8.35 mM DMSO stock, with a starting concentration of 50 μM followed by 2-fold serial dilutions. Fullerene samples were prepared *via* 20-fold dilution of the aqueous stocks into the RdRp assay buffer. All incubation, centrifugation, and measurement parameters (80% LED, 40% MST power) remained identical to the PLpro protocol. Data were analyzed using MO.Affinity Analysis 2.3 and GraphPad Prism 10.

Spike protein binding affinity assays

The binding affinities of fullerenes and naphthofluorescein toward the 2019-nCoV Spike protein (SinoBiological) were determined as described for RdRp, with the following modifications. The assay was conducted in PBS buffer supplemented with 0.05% Tween 20. All other experimental parameters, including His-Tag labeling, centrifugation, and MST measurement settings (80% LED, 40% MST power), were identical to those described in the previous sections. Data were processed using MO.Affinity Analysis 2.3 and GraphPad Prism 10.

3CL-Mpro protein binding affinity assays

The binding affinities of fullerenes and nirmatrelvir toward 3CL-Mpro recombinant protein (BPS Bioscience) were determined as described for PLPro, utilizing the same Protein Labeling Kit RED-NHS 2nd Generation (lysine-specific labeling). The assay was performed in a buffer

consisting of 50 mM Tris-HCl (pH 7.39), 200 mM NaCl, 0.05% Tween 20, 0.1% BSA, and 1 mM EDTA. Nirmatrelvir served as the reference inhibitor, prepared from an 8.35 mM DMSO stock with a starting concentration of 200 nM, followed by 2-fold serial dilutions. All other procedures, including incubation (15 min), centrifugation, and MST measurement settings (80% LED, 40% MST power), were consistent with the protocols described above. Data analysis was conducted using MO.Affinity Analysis 2.3 and GraphPad Prism 10.

Cell culture

The lung adenocarcinoma A549 was purchased from ATCC. The cells were cultured in Dulbecco's modified Eagle's medium (DMEM) that had been supplemented with 10% heat-inactivated fetal bovine serum – FBS (all from Merck) and a mix of two antibiotics - penicillin and streptomycin (1% v/v; Gibco). Cell lines were cultured at 37 °C with 5% CO_2 humidified atmosphere. Cell lines were routinely tested for *mycoplasma* contamination using the PCR technique.

Cytotoxicity studies

The cells were seeded in 96-well plates (Nunc) at a density of 5,000 cells per well and incubated under standard condition at 37 °C for 24 h. The assay was carried out following a 72 h incubation with the various concentrations of the tested nanomaterials. Then, DMEM without phenol red with CellTiter 96®AQueous One Solution-MTS (Promega) solution was added to each well and incubated for 1 h at 37 °C. The optical densities of the samples were measured at 490 nm using a multi-plate reader (Varioskan LUX, Thermo Scientific). The obtained results were compared to the control. Each individual compound was tested in triplicate in a single experiment with each experiment being performed three or four times.

Immunocytochemistry and confocal imaging

A549 cells were seeded onto glass coverslips at a density of 4×10^4 cells per coverslip and cultured for 48 h under standard cell culture conditions. Subsequently, cells were treated with C_{60}Trp at 1 mg/mL for 24 h. Control cells were cultured under identical conditions but were not exposed to the fullerene derivative. Following treatment, mitochondria were stained with MitoTracker Orange CMTMRos (70 nM) for 30 min under standard culture conditions. Cells were then washed three times with phenol red-free DMEM and fixed with 4% paraformaldehyde (PFA) in PBS for 1 h at room temperature (RT). After fixation, samples were washed three times for 5 min each with PBS and permeabilized with 0.1% Triton X-100

in PBS for 15 min at RT. Cells were then washed again three times for 5 min with PBS. Non-specific binding was blocked with 5% bovine serum albumin (BSA) in PBS for 1 h at RT on a rocking platform. After blocking, cells were incubated overnight at 4°C on a rocker with the primary anti-fullerene antibody (Fullerene Antibody, clone 1-10F-A8). The following day, samples were washed three times for 5 min with blocking buffer and incubated with Alexa Fluor 488-conjugated anti-mouse IgG secondary antibody for 2 h at RT. After secondary antibody incubation, samples were washed three times for 5 min with blocking buffer, then mounted with ProLong™ Gold Antifade Mountant containing DAPI.

Images were acquired using a Leica Stellaris 8 confocal microscope equipped with a white light laser (WLL). Fluorescence signals were collected using the following excitation and emission settings: DAPI (excitation: 405 nm, emission: 415–480 nm), Alexa Fluor 488 (excitation: 488 nm, emission: 500–550 nm), and MitoTracker Orange CMTMRos (excitation: 561 nm, emission: 570–620 nm). Z-stack images were acquired and processed to obtain central optical sections and maximum intensity projections (MIPs).

Computational methodology

Protein Preparation. The three-dimensional structures of SARS-CoV-2 Papain-like protease (PLpro, PDB: 7CMD), RNA-dependent RNA polymerase (RdRp, PDB: 7OVZ), 2019-nCoV spike glycoprotein (PDB: 7FCD), and SARS-CoV-2 3CL main protease (3CL-Mpro, PDB: 8DZ2) were retrieved from the RCSB Protein Data Bank. Each structure was prepared for computational analysis by removing all co-crystallized ligands, metal ions, water molecules, and other non-protein heteroatoms. Hydrogen atoms were added and protonation states were assigned at physiological pH (7.4). Protein topology and force field parameters were assigned using the ff14SB force field as implemented in AMBER16.[1, 2]

Fullerene Derivative Parameterization. Four fullerene-based compounds ($C_{60}(OH)_{24}$, C_{60}-Ser, C_{60}-Ala, and C_{60}-Trp) were parameterized following the standard AMBER protocol. Molecular geometries were first optimized at the Hartree-Fock level of theory using the 6-31G* basis set as implemented in Gaussian16. Partial atomic charges were subsequently derived via Restrained Electrostatic Potential (RESP) fitting on the HF/6-31G* electrostatic potential surface, using the *antechamber* module of AmberTools23[3]. Force field bonded and non-bonded parameters were assigned using the General AMBER Force Field 2 (GAFF2)[4]. The resulting topology and coordinate files were validated prior to docking and MD simulations.

Molecular Docking. Molecular docking of all four fullerene derivatives against PLpro (PDB: 7CMD), RdRp (PDB: 7OVZ), spike glycoprotein (PDB: 7FCD), and 3CL-Mpro (PDB: 8DZ2) was performed using AutoDock Vina.[5, 6] For each protein target, the docking search space was defined as a grid box centered on the experimentally characterized binding pocket, with dimensions set to encompass the full binding site with adequate margin. Docking calculations were carried out in triplicate using an exhaustiveness value of 32 and an energy range of 100 kcal/mol. Docking results were ranked according to the predicted binding affinity and the most favorable pose from each triplicate run was retained for subsequent analysis.

Molecular Dynamic simulations. The best-ranked C_{60}-Trp@Spike protein docking pose was solvated in an explicit TIP3P water box with a minimum distance of 20 Å between the solute and the periodic box boundaries. Sodium and chloride counter-ions were added to neutralize the system charge. Prior to dynamics, the solvated system underwent energy minimization in two sequential phases, for a total of 10,000 steps. The first 5,000 steps employed the steepest descent algorithm to remove large steric clashes, followed by 5,000 steps with the conjugate gradient algorithm to refine the geometry to a local energy minimum. Following minimization, the system was equilibrated for 1 ns under constant volume and temperature (NVT) conditions. The temperature was gradually raised from 0 K to 300 K using a Langevin thermostat, allowing the solvent and counter-ions to relax around the solute prior to the production run. Production MD simulations were carried out for 100 ns under periodic boundary conditions (PBC) using AMBER16. Long-range electrostatic interactions were treated with the Particle Mesh Ewald (PME) method, and a cutoff of 10 Å was applied to non-bonded interactions. Bonds involving hydrogen atoms were constrained using the SHAKE algorithm, permitting a 2 fs integration timestep. Atomic coordinates were saved at regular intervals throughout the trajectory for subsequent analysis. The binding free energy of C_{60}-Trp to the spike glycoprotein was estimated using the Molecular Mechanics–Generalized Born Surface Area (MM-GBSA) method, as implemented in AmberTools23[7]. MM-GBSA calculations were performed on each saved frame of the production trajectory to monitor the evolution of binding energetics over the course of the simulation and to identify the most stable bound conformation. MM-GBSA per-residue energy decomposition was performed on the lowest-energy trajectory frame to identify the spike glycoprotein residues contributing most favorably to the interaction with C_{60}-Trp. Residues exhibiting the largest energetic contributions were considered key determinants of molecular recognition and potential targets for further rational design.

Results

			Size (d.n...	% Intensity:	St Dev (d.n...
Z-Average (d.nm):	135,0	Peak 1:	146,5	100,0	43,69
PdI:	0,065	Peak 2:	0,000	0,0	0,000
Intercept:	0,881	Peak 3:	0,000	0,0	0,000
Result quality	Good				

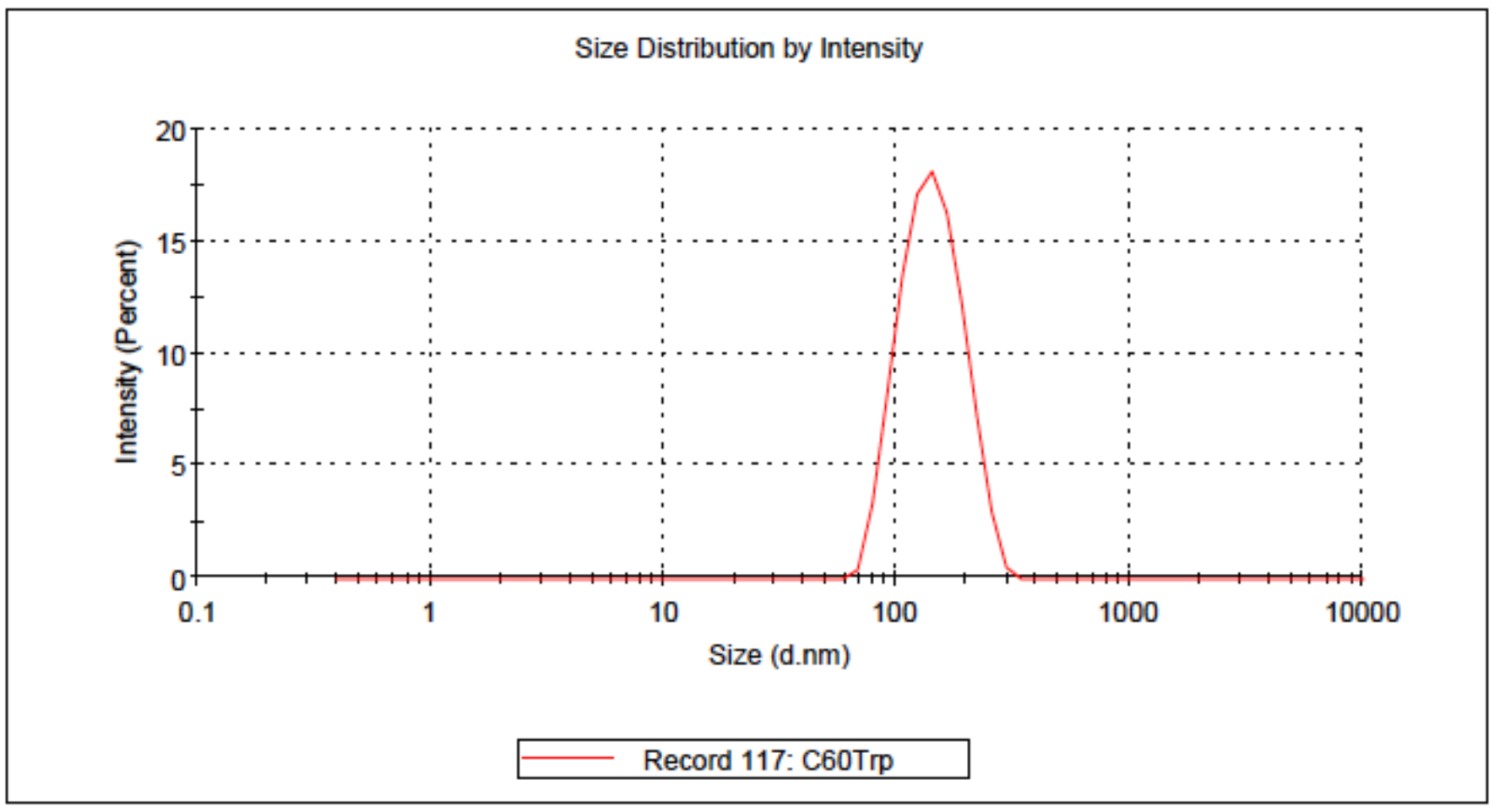

Figure S1

The DLS size measurement of C_{60}Trp fullerene in DI water (c=0.01 mg/mL).

Results

			Mean (mV)	Area (%)	St Dev (mV)
**Zeta Potential (mV):**	-45,5	**Peak 1:**	-45,5	100,0	5,14
**Zeta Deviation (mV):**	5,14	**Peak 2:**	0,00	0,0	0,00
**Conductivity (mS/cm):**	0,0188	**Peak 3:**	0,00	0,0	0,00
**Result quality**	Good				

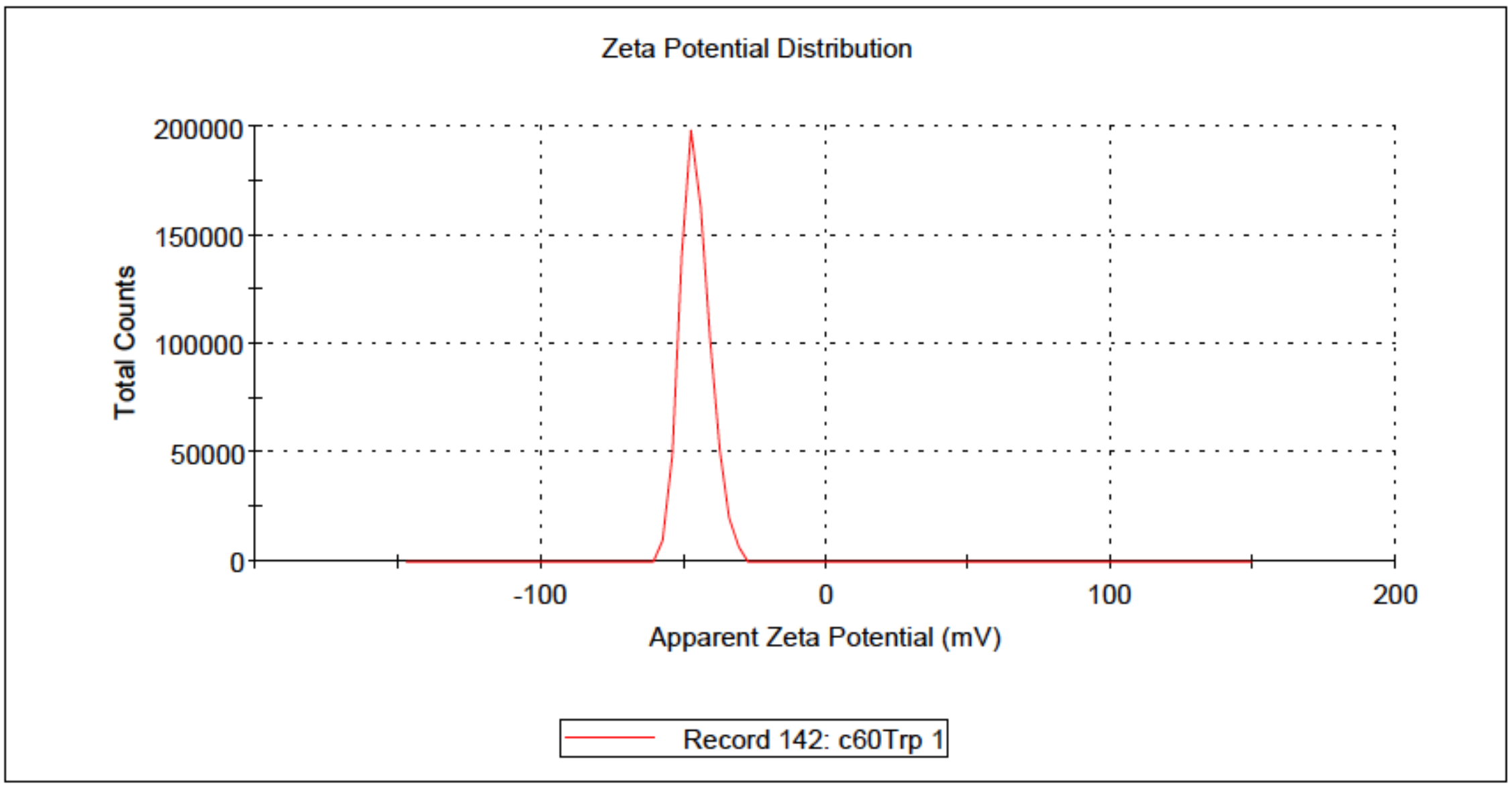

Figure S2

Zeta potential measurement for C_{60}Trp fullerene in DI water (c=0.01 mg/mL).

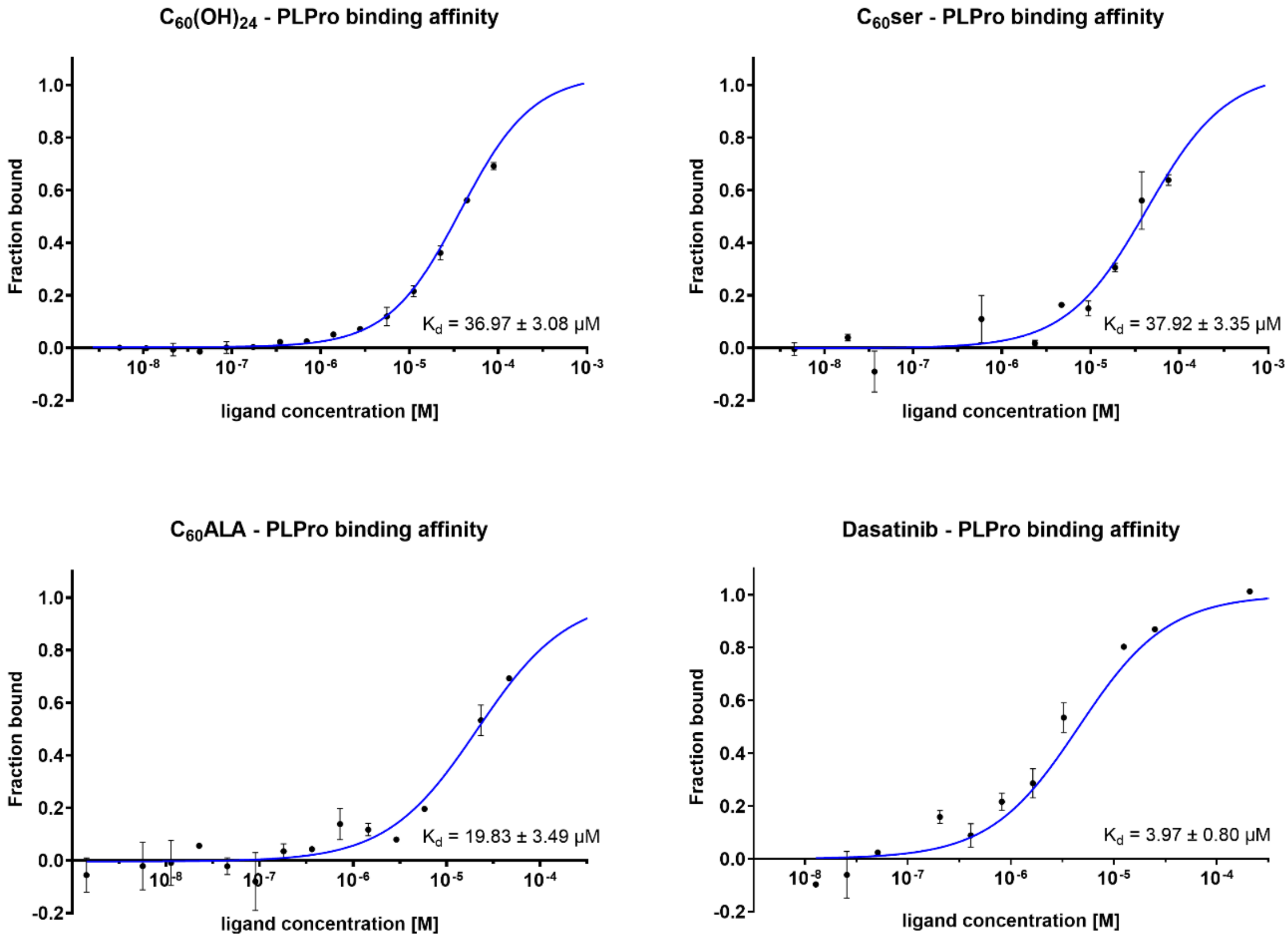

Figure S3

Binding affinity curves of the tested fullerenes and dasatinib toward PLPro protein. The fraction bound is plotted against the ligand concentration on a logarithmic scale. Each data point represents the mean ± SD (n = 3).

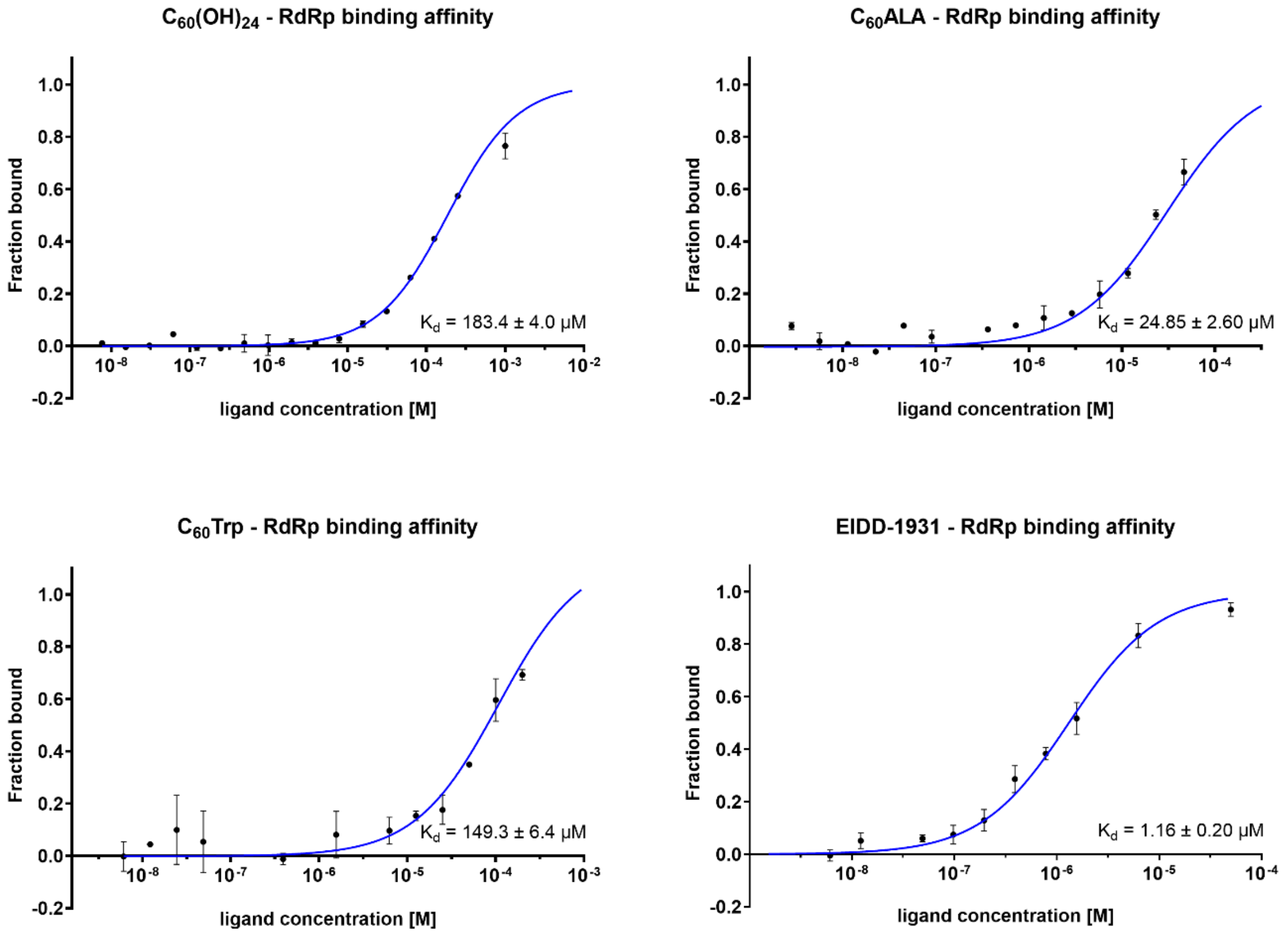

Figure S4

Binding affinity curves of the tested fullerenes and EIDD-1931 toward RdRp protein. The fraction bound is plotted against the ligand concentration on a logarithmic scale. Each data point represents the mean ± SD (n = 3).

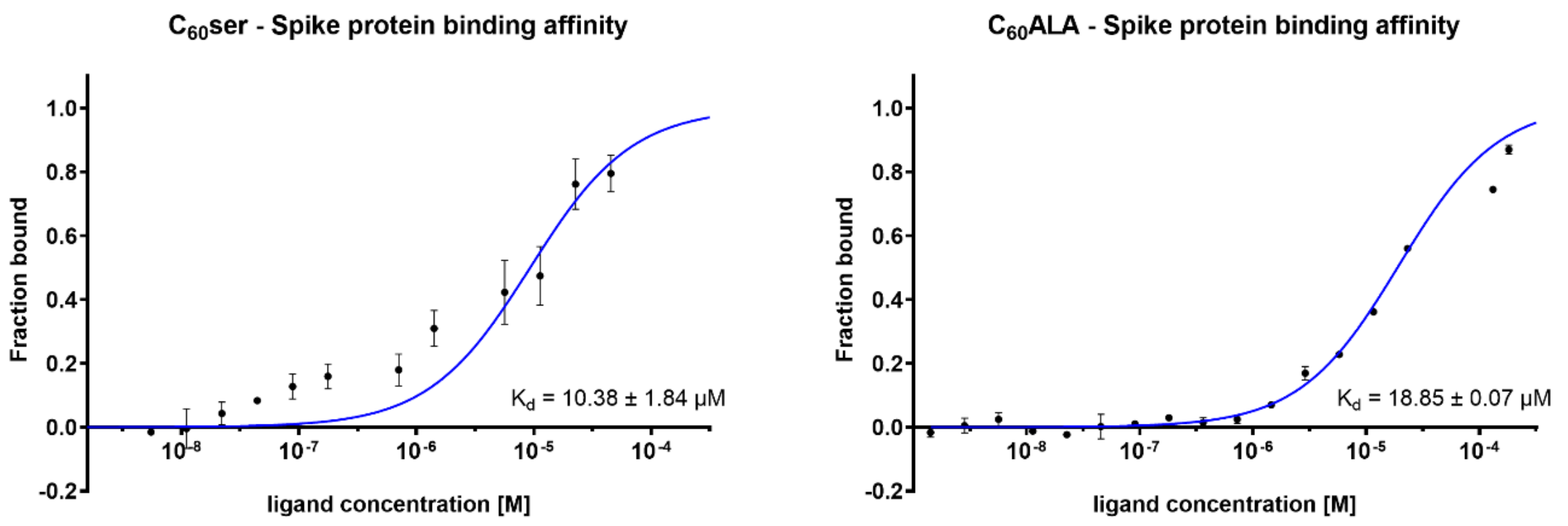

Figure S5

Binding affinity curves of the tested fullerenes toward Spike protein. The fraction bound is plotted against the ligand concentration on a logarithmic scale. Each data point represents the mean ± SD (n = 3).

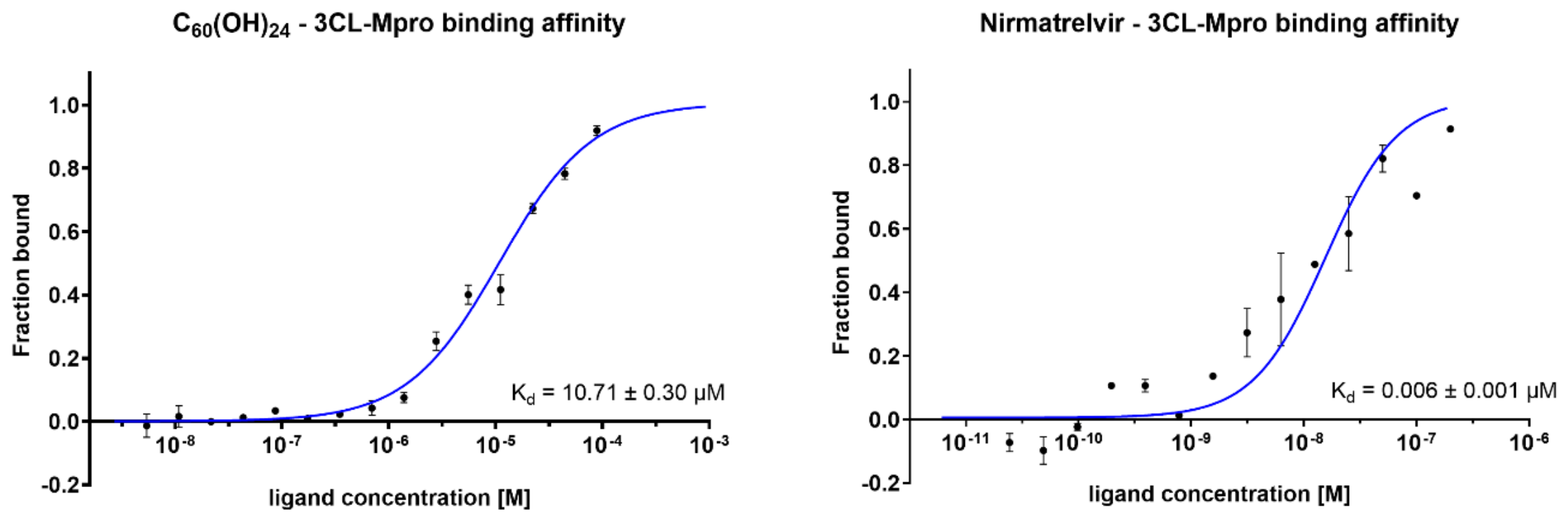

Figure S6

Binding affinity curves of the tested fullerenes and nirmatrelvir toward 3CL-Mpro protein. The fraction bound is plotted against the ligand concentration on a logarithmic scale. Each data point represents the mean ± SD (n = 3).

REFERENCES

(1) Maier, J. A.; Martinez, C.; Kasavajhala, K.; Wickstrom, L.; Hauser, K. E.; Simmerling, C. Ff14SB: Improving The Accuracy Of Protein Side Chain And Backbone Parameters From Ff99SB. *Journal of chemical theory and computation* **2015**, *11* (8), 3696–3713.

(2) Case, D.; Berryman, J.; Betz, R.; Cerutti, D.; Cheatham III, T.; Darden, T.; Duke, R.; Giese, T.; Gohlke, H.; Goetz, A. AMBER 2018. University of California, San Francisco (2018). **2015**.

(3) Cornell, W. D.; Cieplak, P.; Bayly, C. I.; Kollman, P. A. Application of RESP charges to calculate conformational energies, hydrogen bond energies, and free energies of solvation. *Journal of the American Chemical Society* **2002**, *115* (21), 9620–9631.

(4) Wang, J.; Wolf, R. M.; Caldwell, J. W.; Kollman, P. A.; Case, D. A. Development and testing of a general amber force field. *Journal of computational chemistry* **2004**, *25* (9), 1157–1174.

(5) Eberhardt, J.; Santos-Martins, D.; Tillack, A. F.; Forli, S. AutoDock Vina 1.2. 0: new docking methods, expanded force field, and python bindings. *Journal of chemical information and modeling* **2021**, *61* (8), 3891–3898.

(6) Trott, O.; Olson, A. J. AutoDock Vina: improving the speed and accuracy of docking with a new scoring function, efficient optimization, and multithreading. *Journal of computational chemistry* **2010**, *31* (2), 455–461.

(7) Miller III, B. R.; McGee Jr, T. D.; Swails, J. M.; Homeyer, N.; Gohlke, H.; Roitberg, A. E. MMPBSA. py: an efficient program for end-state free energy calculations. *Journal of chemical theory and computation* **2012**, *8* (9), 3314–3321.
